# Ovarian masses in children and adolescents in China: analysis of 203 cases

**DOI:** 10.1186/1757-2215-6-47

**Published:** 2013-07-04

**Authors:** Hongqian Liu, Xiangao Wang, Donghao Lu, Zhihong Liu, Gang Shi

**Affiliations:** 1 Department of Obstetric and Gynecologic, West China Second University Hospital, Sichuan University, Chengdu 610041, China; 2 Laboratory of Cell and Gene Therapy, West China Institute of Women and Children's Health, West China Second University Hospital, Sichuan University, Chengdu 610041, China; 3 Laboratory of Genetics, West China Institute of Women and Children's Health, West China Second University Hospital, Sichuan University, Chengdu 610041, China; 4 Key Laboratory of Obstetric & Gynecologic and Pediatric Diseases and Birth Defects of Ministry of Education, Chengdu 610041, China; 5 Department of Diagnostic Imaging, Affiliated Hospital of Zunyi Medical Collage, 201 Dalian Road, Zunyi, Guizhou 563003, China; 6 Department of Medical Epidemiology and Biostatistics, Karolinska Institutet, Stockholm 17177, Sweden

**Keywords:** Ovarian mass, Pediatric, Germ cell, Epithelial tumor, Laparoscope

## Abstract

**Objective:**

The true incidence of ovarian tumors in children is unknown. Few studies beyond case reports and case series have been published concerning pediatric ovarian tumors. Herein we review a large number of ovarian tumor cases.

**Methods:**

The charts of 203 patients who presented with adnexal masses were reviewed.

**Results:**

The patient’s ranged in age from 2 to 18 years (mean = 15.6 years), with 30 being premenarchal (14.8%). The incidence of ovarian tumor increases with age, especially in patients older than 14 years. The main complaint was abdominal pain or abdominal distension in 117 patients (57.7%). A high AFP level in a pre-pubic girl with an adnexal mass is indicative of a malignant ovarian tumor. The 214 adnexal masses (11 patients had bilateral cysts) consisted of benign tumorous oophoropathy (107 masses, 50.0%), borderline and malignant tumors (29 masses, 13.6%), and nontumorous oophoropathy (78 masses, 36.5%). Of the 136 neoplasia, germ cell tumors accounted for 71.5%. Surgical intervention was performed in 98.5% of cases. There were statistically decreased blood loss, surgery duration and days of hospitalization with the laparoscopic procedure when compared with open surgery.

**Conclusions:**

Abdominal pain is the most common complaint in young patients with adnexal masses. AFP is the most useful diagnostic biomarker of ovarian tumors in young females. Laparoscopic resection of ovarian cysts is an alternative operation approach.

## Introduction

Ovarian masses are classified as non-neoplastic and neoplastic. Functional cysts, ovarian torsion, and benign neoplasms are the most common ovarian masses in young adolescents. Germ cell malignant tumors are the most frequent malignant tumors of childhood. This contrasts with adults, in whom epithelial malignant tumors account for most malignant ovarian neoplasms. Although malignant ovarian neoplasms constitute a small proportion of all tumors in children and adolescents (less than 2%), they are the most common pediatric gynecologic tumor [1,2]. Malignant ovarian tumors in children and adolescents are rare, accounting for 0.9% of all malignancies [3]. In premenarchal girls, up to 40% of ovarian neoplasms are malignant [4]. However, the true incidence of malignant ovarian tumors in the pediatric population is unknown, as few studies aside from case reports and case series have been published.

The diagnosis of an ovarian mass is typically determined by surgical and histological means. Laparoscopic surgery is the accepted gold standard for management of adnexal masses. Indeed, benign ovarian neoplasms are treated via laparotomy by many gynecologists and pediatric surgeons. In cases of malignancy, the recent literature is controversial. Mayer et al. reported one case of peritoneal implantation of squamous cell carcinoma following rupture of a dermoid cyst during laparoscopic removal [5]. All surgical procedures for treatment of ovarian cysts should spare the functional ovary as far as is technically possible. Kathleen et al. have suggested that the surgeon should implement ovarian-sparing strategies on the affected ovary (unless a malignancy is clearly suspected) and conserve the contralateral ovary in all cases [6].

Females under the age of 18 with ovarian masses are unique in terms of clinical symptoms, pathological subtypes and the treatment required. The most recent study focusing on children with adnexal masses (240 cases) was published in 2003 [7]. In this report, we provide an overview of 203 young female patients treated for adnexal masses in our hospital over the last three years. The epidemiology, clinical features, and treatment of this unusual pathology are discussed.

## Methods

We reviewed the records of 203 females under the age of 18 years with adnexal masses who were treated at the West China Second Hospital (Sichuan, China) from 2007 to 2009. Patient demographics and clinical characteristics were extracted from the hospital database with approval of the Institutional Review Board. All patients underwent pelvic ultrasound. Tumor markers, including serum alpha- fetoprotein (AFP), beta-human chorionic gonadotropin (β-HCG), cancer antigen 125 (CA-125), carbohydrate antigen 19–9 (CA 19–9) and carcinoembryonic antigen (CEA), were tested in most cases. The stages of malignant ovarian tumors were classified according to FIGO guidelines. Patients were followed-up for an average of 17 months (range: 1 to 6 months for benign masses; 1 month to 3 years for malignant neoplasms). Patients for whom data were missing data were excluded from analysis. We conducted the covariate analysis to compare the operation time, operation bleeding and length of hospital stay between two groups when adjusted the tumor size, with a threshold of significance of a probability value of ≤ 0.05. We used the SPSS 16.0 software for all statistics analysis.

## Results

203 patients with ovarian tumors were identified. Patients under 18 years of age accounted for 2.96% of all female patients with adnexal masses over the three year period. The breakdown of cases included 3.4% ≤9 years of age, 26.2% between 10 and 14 years of age, and 70.4% between 15 and 18 years of age. The mean patient age was 15.6 years (range: 2 to 18 years). 64% of patients were rural dwellers and 36% urban dwellers. 30 patients (14.8%) were premenarche, while the mean age of menarche was 12.5 ± 1.8 years. 32 patients (15.8%) described having sexual encounters, with 5 having a history of pregnancy (these included one patient who had a hydatidiform mole and two who experienced ectopic pregnancy). 24 patients (11.8%) were emergency admissions, while 179 (88.2%) were routine admissions. All patients were Han Chinese.

**Figure 1:**
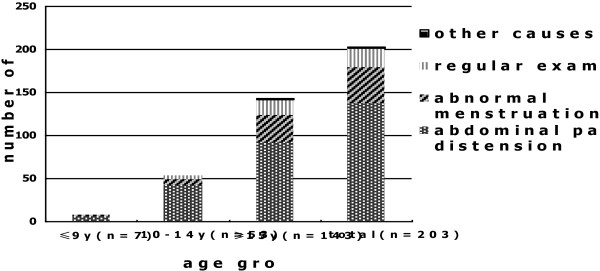
Main complaint by age grouping.

The main complaint was abdominal pain or abdominal distension of several months duration, with or without increasing severity in 117 patients (57.7%). Of these patients, 23 (19.7%) were emergency admissions. 41 patients (20.2%) presented with abnormal menstruation, including menstruation irregularis, menopause and dysmenorrhea. 22 adnexal masses (10.8%) were identified by health examination. The distributions of the chief complaints for the different age groups are shown in Figure 1.

Tests for serum tumor makers including AFP, CA125, CA199, CEA, and HCG were performed in most cases. No patients with nontumorous masses or benign tumors had abnormal serum AFP or CEA levels. Of the patients with malignant tumors, 73.7% had abnormal AFP and CA125 levels. Nearly 20% of patients with nontumorous masses and benign tumors also had abnormal CA125 levels. These findings are summarized in Table 1.

**Table 1:** Results of serum tumor makers

	**Non tumorous mass**			**Benign ovary tumor**			**Malignant ovary tumor**		
	**Tested**	**Positive**		**Tested**	**Positive**		**Tested**	**Positive**	
	**No.**	**No.**	**(%)**	**No.**	**No.**	**(%)**	**No.**	**No.**	**(%)**
**AFP**	31	0	0	66	1	1.52	19	14	73.68
**CEA**	20	0	0	43	0	0	14	2	14.29
**CA125**	39	9	23.08	75	14	18.67	18	12	66.67
**CA199**	31	6	19.35	54	24	44.44	15	6	40
**HCG**	12	4	33.33	14	1	7.14	7	5	71.43

Of the 214 adnexal masses (11 patients had bilateral cysts), there were 107 benign tumorous oophoropathies (50.0%), 29 borderline and malignant tumors (13.6%), and 78 nontumorous oophoropathies (36.5%). Approximately 10% of patients had ovarian torsion. The mean adnexal mass volume was 8.93 ± 6.43cm (range: 2 to 40cm). The 136 neoplasias consisted of germ cell tumors (72.1%), epithelial tumors (22.8%), and sex cord-stromal tumors (5.1%). Nontumorous tumors included parovarian cysts, corpus luteal cysts, ovarian endometriosis cysts, hemorrhagic or follicular cysts, paratubal cysts, hydrosalpinx and adnexal abscess. The classification and distribution of tumors are summarized in Table 2 with respect to patient age.

**Table 2:** Histologic distribution of 203 cases of adnexal masses of children and adolescent by age of patient

	**Age of patient**							
	**≤9 y**		**10–14 y**		**≥15 y**		**total**	
	**No.**	**%**	**No.**	**%**	**No.**	**%**	**No.**	**%**
Germ cell tumor	**5**	5/7 (83.33%)	34	34/54 (62.96%)	59	59/153 (38.56%)	98	98/214 (45.79%)
				**34/37 (91.89%)**		**59/92 (64.13%)**		**98/136 (72.06%)**
Mature teratoma	4		31		50		85	
Immature teratoma	1		2		2		5	
Endodermal sinus tumor	0		1		5		6	
Mix germ cell tumor					2		2	
Epithelial tumor	0	0	2	2/54 (3.70%)	29	29/153 (18.95%)	31	31/214 (14.49%)
				**2/37 (5.41%)**		**29/92 (31.52%)**		**31/136 (22.79%)**
Mucinous tumor	0		2		13		15	
Benign	0		2		7		9	
Borderline	0		0		2		2	
Malignant	0		0		4		4	
Serous tumor	0		0		15		16	
Benign	0		0		11		11	
Borderline	0		0		4		4	
Malignant	0		0		0		0	
Sex cord-stromal tumor	2	2/7 (16.67%)	1	2/54 (1.85%)	4	4/153 (2.61%)	7	7/214 (3.27%)
				**2/37 (2.70%)**		**4/92 (4.35%)**		**7/136 (5.15%)**
Granular cell tumor	1		0		0		1	
Steroid cell tumors	0						2	
Well differentiated					1			
malignant			1					
Dysgerminoma	1		0		3		4	
**Nontumorous mass**	**0**	**0**	**17**	**17/54 (31.48%)**	**61**	**61/153 (39.87%)**	**78**	**78/214 (36.45%)**
Parovarian cyst	0		1		18		19	
Lutein cyst	0		12		19		31	
other	0		4		24		28	
Total	7	7/214 (3.27%)	54	54/214 (25.23%)	153	153/214 (71.50%)	214	100

Numbers in boldface represent percentage relative to 136 ovarian tumors.

Numbers in parentheses represent percentage relative to 136 ovarian tumors.

The percentage of patients who underwent operative intervention was 98.5%. Only 3 patients did not receive surgery; one experienced kidney failure six months after endodermal sinus tumor surgery, while the two other had luteal cysts and received conservative treatment. 117 laparoscopic procedures were performed; two of these progressed to open surgery. Ovarian lesions were associated with ovarian torsion (20), mature teratomas (13), cystadenomas (2), and nontumorous masses (4). There was no pathological explanation for one lesion. 11 of 20 ovarian torsions were managed conservatively, while ovariectomy was reserved for non-resectable tumors and gangrenous necrosed ovaries that did not bleed upon incision 15 minutes after detorsion. There were significant difference between laparoscopic group and open group after adjustment of tumor size. The analysis results about two therapies for benign adnexal masses are shown in Table 3.

**Table 3:** Comparison of two therapies for benign adnexal masses

	**Opertation time**	**Operation bleeding**	**Length of hospital stay**
	**(Mean ± SE)**	**(Mean ± SE)**	**(Mean ± SE)**
Open group(n = 34)	79.73 ± 4.59	92.10 ± 7.44	6.93 ± 0.24
Laparoscopic group(n = 117)	60.46 ± 3.11	28.92 ± 5.06	3.78 ± 0.16
P value	0.0008	<0.0001	<0.0001

Conservative surgery includes oophorocystectomy and unilateral oophorectomy. Oophorocystectomy was the most common procedure performed in 81 per cent of cases. The remaining 18 percent underwent unilateral oophorectomy. 21 patients with malignant ovarian tumors and 6 with borderline ovarian tumors received conservative surgery, ten had omentectomy, and two had contralateral ovarian bivalving or biopsies performed; only two underwent radical surgery due to ovarian mucoid adenocarcinomas (stage III). Only three patients underwent laparoscopic surgery. 8 of 23 patients had stage I tumors. Adjuvant chemotherapy following surgery was administered in 6 cases (3 courses of velbe bleomycin, cysplatinum over 3 months). There was no evidence of recurrence over a mean of 17 months follow-up. Normal menstruation resumed after surgery and chemotherapy in 15 patients who had fertility-sparing treatment for malignant ovarian germ cell tumors. One patient with an endodermal sinus tumor (volume = 40 cm, stage III), had high levels of serum tumor biomarkers (AFP >1000 ng/ml, CA125 >600 U/ml). This patient underwent unilateral adnexectomy, during which she lost 15000 ml of blood. The patient subsequently chose to discontinue treatment and died of renal failure three months after surgery.

## Discussion

Adnexal masses may originate from the ovaries, fallopian tubes and (or) other pelvic organs. The types of adnexal masses include tumors, or inflammatory or functional cysts. Approximately one third of adnexal masses are ovarian tumors. In keeping with findings from other studies, children and adolescents with ovarian tumors constituted a very small number of cases in our institution [1,2]. The incidence of ovarian tumors increased with age, being most common in patients older than 14years. This suggests that prolonged exposure to unknown environmental or internal factors may play a role in promoting the development of these tumors [8]. To this end, sex hormones (the concentrations of which increased after menarche) must be considered.

Abdominal pain is the most common symptom in young patients with adnexal masses. Ultrasound should be performed in young females presenting with such symptoms to identify or rule out the presence of adnexal masses. However, abdominal pain is an atypical symptom in 30 to 50% of young females, leading to misdiagnosis. Moreover, nearly 1 out of 5 peri-pubertal patients complained of having irregular menstrual cycles without any pain. A small proportion of patients had no symptoms. Hence specific care should be paid when performing regular physical examinations of children and adolescents.

The gold standard for diagnosing ovarian masses is ultrasound [3]. Malignant tumors are complex soft-tissue masses with ill-defined irregular borders and central necrosis, septations, and papillary projections. AFP and CA125 are the most useful biomarkers for distinguishing patients with malignant tumor in young females. Normal tumor markers levels, namely AFP and CA125, are suggestive of a benign cyst. A high level AFP level in a pre-pubic girl with an adnexal mass is indicative of a malignant ovarian tumor. However, in general, for children CA-125 is of limited utility, as it will not affect the indication for surgical exploration of persistent masses [9], which is coincident with our review.

Ovarian torsion is a rare problem that must be considered in the differential diagnosis of any pediatric female patient presenting with abdominal pain or a pelvic or abdominal mass. Continued arterial perfusion often leads to ovary enlargement and subsequent necrosis, infarction, and local hemorrhage. This in turn may lead to peritonitis, and in some cases systemic infection and inflammation. Therefore, it is essential to diagnose and treat ovarian torsion as early as possible to preserve the uterine adnexa. Emergency ultrasound is warranted, however only surgery, most often via laparoscopy, allows for a definitive diagnosis to be made [10]. In our cohort, around half of patients with ovarian torsion underwent resection of the involved adnexa for scarcity of timely admission. Gynecologists and pediatric surgeons should consider the possibility of ovarian neoplasms in any young female patient presenting with an abdominal mass or abdominal pain regardless of age and despite the fact that such neoplasms are rare in children. Regular physical examinations and appropriate tests are advisable for young patients. Laparoscopy allows for a combination of diagnosis and (if necessary) therapeutic intervention in young patients with symptoms suggestive of ovarian masses.

After reviewing six pediatric cases of unexplained ovarian torsion, Anish and colleagues suggested that early polycystic ovary syndrome (PCOS) may be an underlying cause of unexplained premenarcheal ovarian torsion [11]. In the present cohort of patients there was one case of unexplained ovarian torsion; however there was no evidence of PCOS.

One aim of this study was to summarize our recent experience treating adnexal disease using the laparoscopic approach. Mayer and colleagues previously suggested that laparoscopy (for treatment of ovarian cysts, including those with large dimensions) is associated with satisfactory outcomes and few complications [12]. There is an increased risk of perforation with trocar insertion during laparoscopy for treatment of giant ovarian cysts. These cysts may exceed the working space [13]. Mayer et al. described a laparoscopic procedure in which the giant cyst is punctured and the cyst wall subsequently removed [12]. We successfully performed laparoscopy on a two-year old patient with a unilateral teratoma (diameter = 5cm) with torsion. Laparoscopy is preferred over intraabdominal surgery for treatment of benign masses in children and adolescents due to decreased surgery duration, blood loss and days of hospitalization.

The true incidence of pediatric malignant ovarian tumors is unknown. In the present study, 71.50% of tumors occurred in patients aged between 15 and 18 years. Germ cell tumors were the most common malignant ovarian tumor in our cohort of patients, comprising 72.06% of all tumors. This incidence is apparently higher than that noted in previous reports [1,10,11]. Deprest et al. pooled data for 1364 patients ages 0 to 19 y and reported 62.2% of ovarian tumors were of germ cell histologic type [14]. In a review of 57 cases of ovarian tumors in 0 to 19 year olds, Hassan et al. reported that ovarian sex cord stromal tumors accounted for 12.3% of all tumors [1]. In contrast, we found that 5.14% of pediatric ovarian tumors were sex cord neoplasms. We found the histological distribution of tumors to be different from that previously reported, but similar to that noted in a recent review [6]. The differences may be explained by the fact we used a more recent database of ovarian tumors for analysis. It is possible that the histologic classification of tumors differed between ours and those less recent reports.

In summary, we have presented important descriptive data from a large cohort of patients with pediatric ovarian tumors in China. The incidence of ovarian tumors increased with age, being most common in patients older than 14 years. Abdominal pain is the most common complaint in young patients with adnexal masses. The diagnostic standard for ovarian tumors is ultrasound. AFP is the most useful biomarker for ovarian tumors in young females. Laparoscopic resection of ovarian cysts is an alternative operation approach.

## Competing interests

The authors declare that they have no competing interests.

## Authors’ contributions

HL: study design, clinical studies, data analysis and manuscript preparation. XW: clinical studies, data analysis and manuscript preparation. DL: the guarantor of integrity of the entire study, study design, data analysis and manuscript preparation. ZL: clinical studies and data analysis. GS: study design and manuscript review. All authors read and approved the final manuscript.
